# Supplementary material for: Crawling and Gliding: A Computational Model for Shape-Driven Cell Migration
Source: PLoS Comput Biol. 2015 Oct 21;11(10):e1004280. doi: 10.1371/journal.pcbi.1004280 (PMC4619082; doi:10.1371/journal.pcbi.1004280)
Supplement: S1 Code — (ZIP) [file pcbi.1004280.s012.zip › release/tst/doc/html/hull_8h_source.html]

Tissue Simulation Toolkit: hull.h Source File


Tissue Simulation Toolkit
 0.1.4.1


- Main Page
- Namespaces
- Classes
- Files

- File List
- File Members

hull.h

Go to the documentation of this file.

1 // Class point needed by 2D convex hull code

2 class Point {

3

4 public:

5  Point(float xx, float yy) {

6  x=xx;

7  y=yy;

8  }

9  Point(void) {

10  x=0; y=0;

11  }

12  float x,y;

13

14 };

15

16 int chainHull\_2D( Point\* P, int n, Point\* H );

17

Point

**Definition:** hull.h:2

Point::x

float x

**Definition:** hull.h:12

Point::Point

Point(float xx, float yy)

**Definition:** hull.h:5

chainHull\_2D

int chainHull\_2D(Point \*P, int n, Point \*H)

**Definition:** hull.cpp:37

Point::y

float y

**Definition:** hull.h:12

Point::Point

Point(void)

**Definition:** hull.h:9


---

Generated on Thu Aug 14 2014 22:04:01 for Tissue Simulation Toolkit by  

 1.8.6
